# Supplementary material for: COVID-19 in schools: Mitigating classroom clusters in the context of variable transmission
Source: PLoS Comput Biol. 2021 Jul 8;17(7):e1009120. doi: 10.1371/journal.pcbi.1009120 (PMC8266060; doi:10.1371/journal.pcbi.1009120)
Supplement: S1 Text — Within S1 Text we have: Table A. Parameters for the three gamma-distributed time intervals in our model. Fig A. Distribution of latent period, PIP, and infectious period for our simulations. Fig B. Comparison of model results with analytical results in simplified setting. Fig C. Cluster sizes in a high school. Fig D. Students disrupted in a high school. Fig E. Student-days of undetected infection in a high school. Fig F. Comparison of cluster size in a pre-COVID high school with modified plan. Fig G. Cluster size under different parameter settings. Fig H. Cluster size under further different parameter settings. (PDF) [file pcbi.1009120.s001.pdf]

S1 Supplemental Material for *COVID-19 in schools: mitigating classroom clusters in the context of variable transmission*

Paul Tupper, Caroline Colijn

Distribution of latent period, presymptomatic infectious period (PIP), and infectious period. The variation in these three time intervals are all modeled with a gamma distribution. In the main text we specified the mean μ and the standard deviation σ. Most software packages use a parametrization with shape k and scale θ where $\mu = k\theta$ and $\sigma^2 = k\theta^2$ [1, 2]. Another parametrization is with shape k and rate θ^{-1}. We show these values in Table A. In Fig A we plot histograms of the distributions of these time intervals.

Time interval	Mean	St Dev	Shape k	Scale θ	Rate θ^{-1}	citation
latent period	3	1	9	1/3	3	[3]
PIP	2	0.5	16	1/8	8	[4]
infectious period	10	5	4	2.5	0.4	[5]

Table A. Parameters for the three gamma-distributed time intervals in our model. Mean, standard deviation and scale have units of days, shape is dimensionless and rate has units days^{-1}.

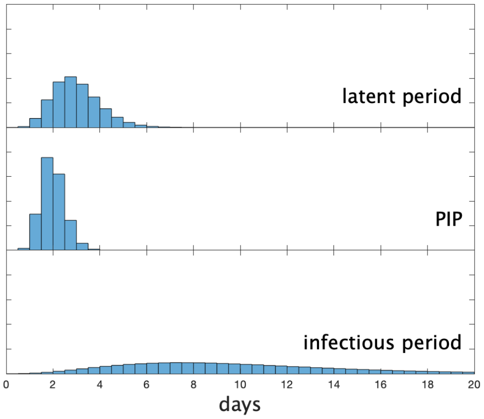

Fig A. Distribution of latent period, PIP, and infectious period for our simulations.

Details of simulation method. Our model tracks the individual state of each of the students in the classroom. Each student is in one of states S (susceptible), E (exposed, not infectious), P (presymptomatic, infectious), Sym (symptomatic infectious, but removed from class), and R (recovered). Latent periods, presymptomatic infectious periods (PIPs), and infectious periods were generated for all students at the start of the simulation whether needed or not. Initially a single student in state P is in the class while all other students are in state S. The states of the students were updated at $\Delta t = 1$ hr time intervals. When students were in school, for each student in state S a total transmission rate is computed by summing over the transmission rate of all students in state P in the class to give a total transmission rate β. The student is then moved

to state E with probability $\beta\Delta t$. At each time step, students are moved from state E to P based on the students' latent periods, from state P to Sym based on the PIPs, and from either P or Sym to R based on the infectious periods. At each time step conditions are checked for whether a flag is set to shut down a group or shut down the whole class. Then a duration of time t_{delay} later, the group or class is shut down, with all transmission being stopped. Similar procedures are used to model pooled testing. See the simulation code available in the repository for full details.

Analytical comparisons in a simplified setting. Our model is sufficiently complex that we cannot obtain analytical results for the distribution of any variables of interest. However, if we make several simplifications to our model, there is a simple form for the distribution of the total number of infected individuals in a classroom. We can then check that the results of our simulations match the results obtained by directly sampling from this distribution.

The first modification we make to our model is to prevent those who are infected by the index case from infecting anyone else in turn. This is achieved by altering one line of code so that those who are infected are moved to the recovered state R, rather than the exposed state E. This is equivalent to only counting the index case and the people they directly infect in the total number of infected individuals. The second modification is to change the presymptomatic infectious period (PIP) and the infectious period to be deterministic rather than random. In each case by altering one line of code we replace the Gamma distributed random variables with their average.

With these modifications the distribution of the number of infected individuals becomes relatively simple. We first consider a symptomatic index case. The index case starts out in a class full of susceptible individuals and remains there until they develop symptoms, which is two days later or $\mu_t = 12$ classroom hours later (2 days $\times$ 6 school hours per day). For each susceptible student in the same group as the index case, there is a rate of transmission $\beta_{\mathrm{in}} = \beta f_{\mathrm{index}}$ where β is either 0.003 hours^{-1} (for environmental β high) or 0.006 hours^{-1} (for environmental β low) and f_{index} is either 1 or 3 (for a higher transmitting index case). The probability of each student in the group being infected is then $p = 1 - \exp(-\beta_{\mathrm{in}}\mu_t)$. Since there are four students in the group other than the index, the number of new infections in the group is binomial with parameters $n = 4$ and p. The number of infections outside the group is computed similarly but with rate $\beta_{\mathrm{out}} = \beta f_{\mathrm{index}} f_{\mathrm{aero}}$ and $n = 20$. We take one (for the index) plus the sum of independent copies of these two binomial random variables to obtain a random variable with the same distribution as the number of infected individuals in our modified simulation code. We denote this distribution by F_{symp}.

When the index case is asymptomatic we perform the same calculation but replace μ_t with the mean infectious duration μ_i (subtracting two days for the weekend) and multiply β_{in} and β_{out} by f_{asymp}. We denote this distribution by F_{asymp}.

In Fig. B we compare results of sampling from F_{asymp} and F_{symp} directly (blue) with the results of our modified code (red). We consider the situation where there are no interventions in the classroom other than symptomatic students being sent home (baseline). There is excellent agreement between the two, the only differences being due to sampling error and the fact that we use a finite (non-zero) time step in our simulations.

Results for high schools. Here we provide the same results for high schools as we did for elementary schools in the main text. For each of the conditions and protocols, Fig C shows total number of infected students in the clusters, Fig D shows the number of students disrupted, and Fig E shows the number of asymptomatic student-days. The effect of the protocols are more modest than in the elementary schools, though this is in large part because the high school structure we consider is already quite good at restricting transmission. The key feature is that transmission is low in the afternoon class because of social distancing (and it only meeting two days a week). The morning class is somewhat larger than the elementary school class, but only meets for half the time.

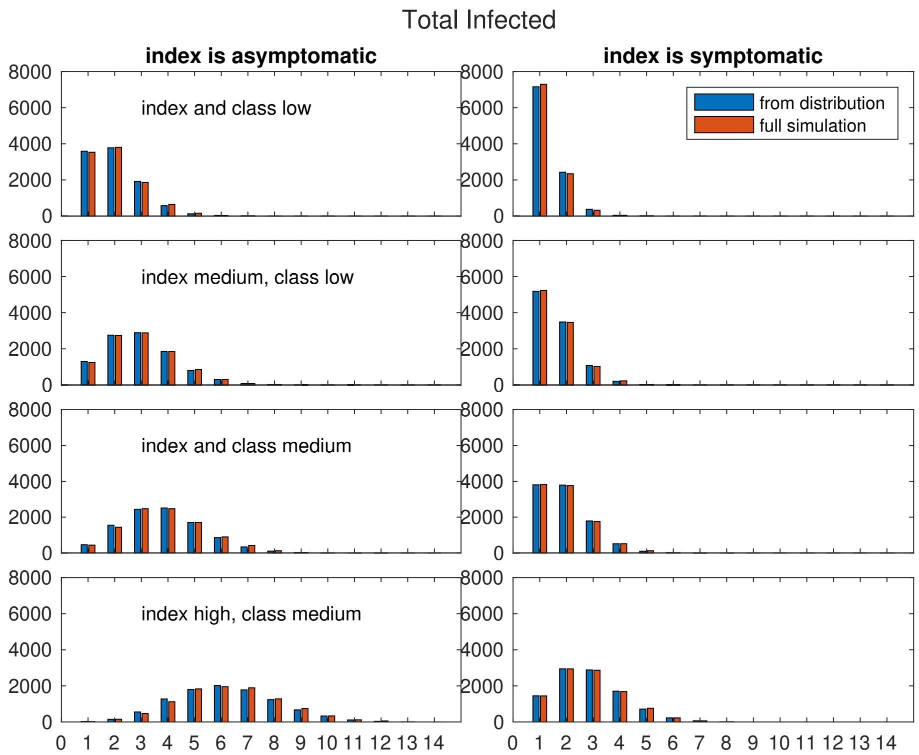

Fig B. Distribution of total infected for four different choices of transmission parameters, and whether the index case is asymptomatic or not. We compare results directly simulated from the analytically known distribution to those obtained from our simulations.

For purposes of illustration we also compare our high school model with a model corresponding to a pre-COVID structure in which students go to 4 different classes every day for 1.25 hours. Fig F shows the difference in cluster size between the two high school structures.

Alternative parameter choices. Figs G and H show the total cluster sizes under different assumptions for the pre-infectious period (PIP) which was mean 2 days (standard deviation 1 day) in the main text, the relative infectiousness of asymptomatic individuals (0.8 times that of symptomatic individuals in the main text) and the extent to which exposure is focused in the identified group of close contacts of an individual. (We took equal numbers of simulations with each of the assumptions on β and the infectiousness of the index case that we used in the main text.) Aerosol transmission and extended mixing in the classroom would both serve to expose individuals who would not be identified as among the close contacts. If (1) the PIP is short, (2) if individuals without symptoms transmit much less than those with symptoms and (3) there is very limited aerosol transmission and very limited mixing outside a known group of close contacts within the classroom, then cluster sizes remain small (though nonzero) no matter the intervention protocol. Assumptions (1)-(3) are all strong and optimistic assumptions, and are not supported by data in adults or by viral load data from asymptomatic individuals. However, if these assumptions did hold in children then school transmission should be rare and would likely involve a high fraction of transmission among teachers and other adult staff when it did occur. In Figs G and H, we progress from left to right columns of panels, first assuming all three of these assumptions and then allowing for a longer PIP, higher asymptomatic transmission and finally higher mixing/aerosol transmission.

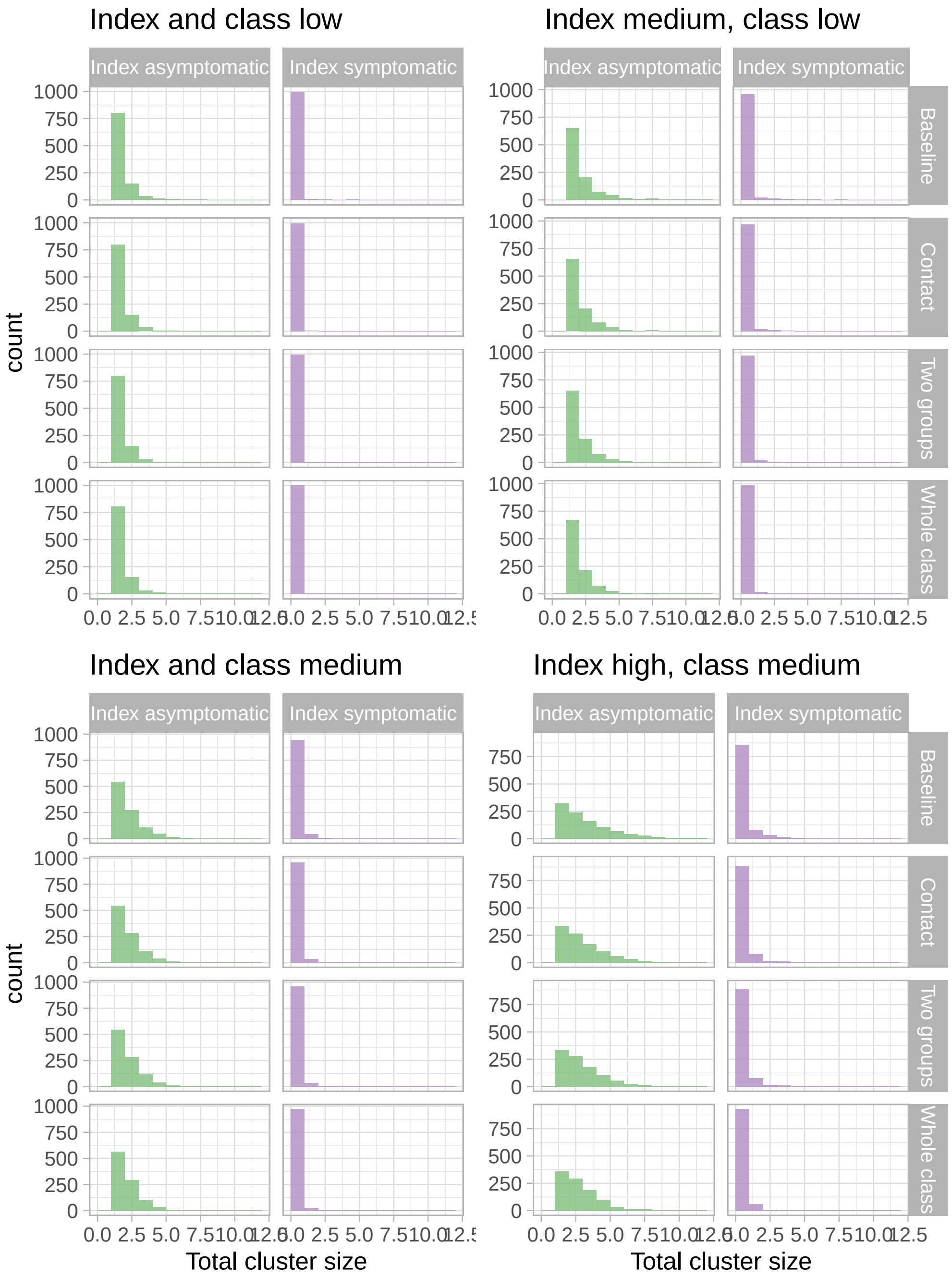

Fig C. High school total cluster sizes are smaller and less variable than those in elementary schools because high schools are operating with one full in-person class every day and another, smaller and distanced, class two afternoons per week. However, if the index case is asymptomatic and high-risk, even this protocol can allow a cluster of 10 infections.

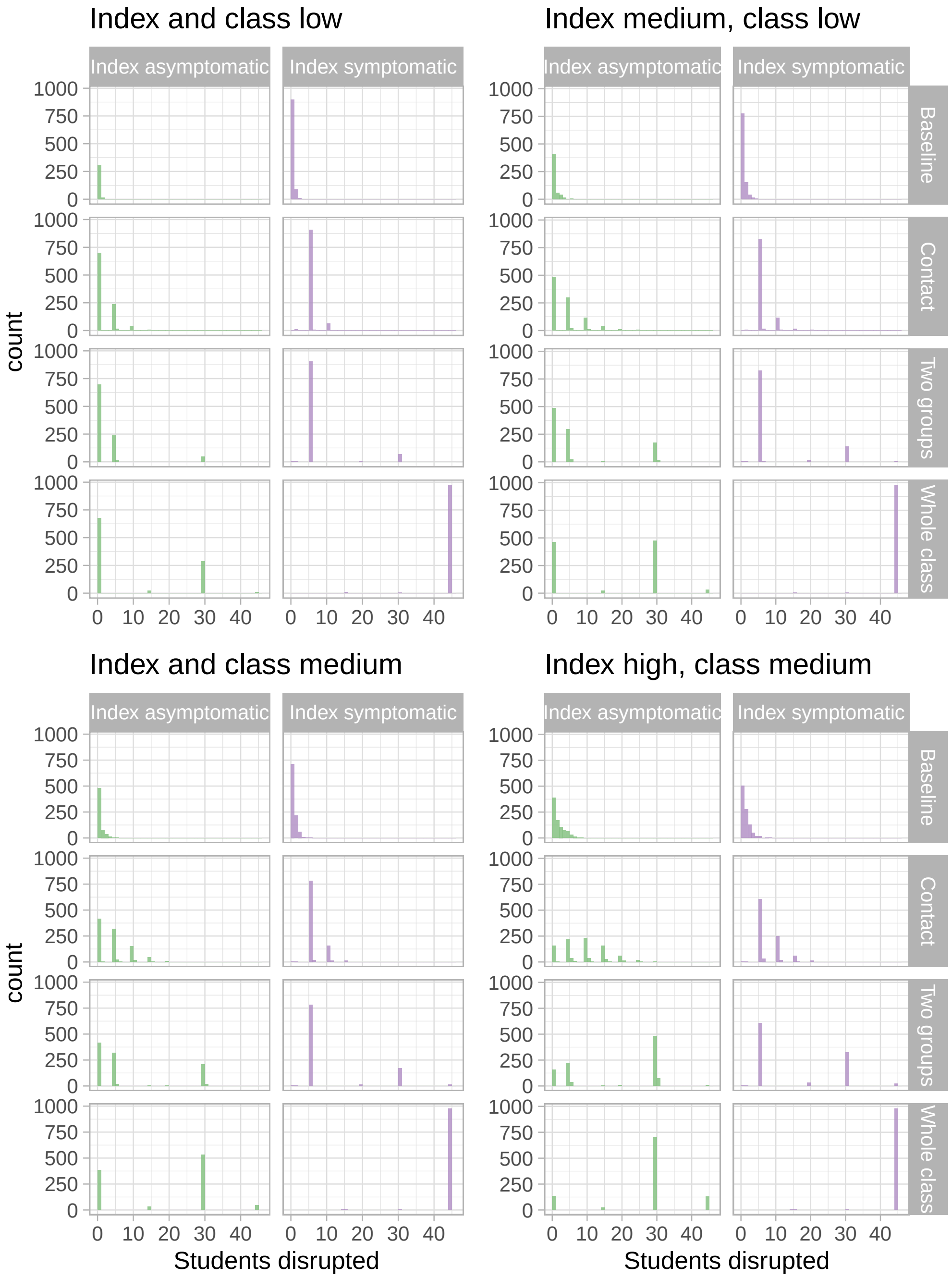

Fig D. Students disrupted in the high school model.

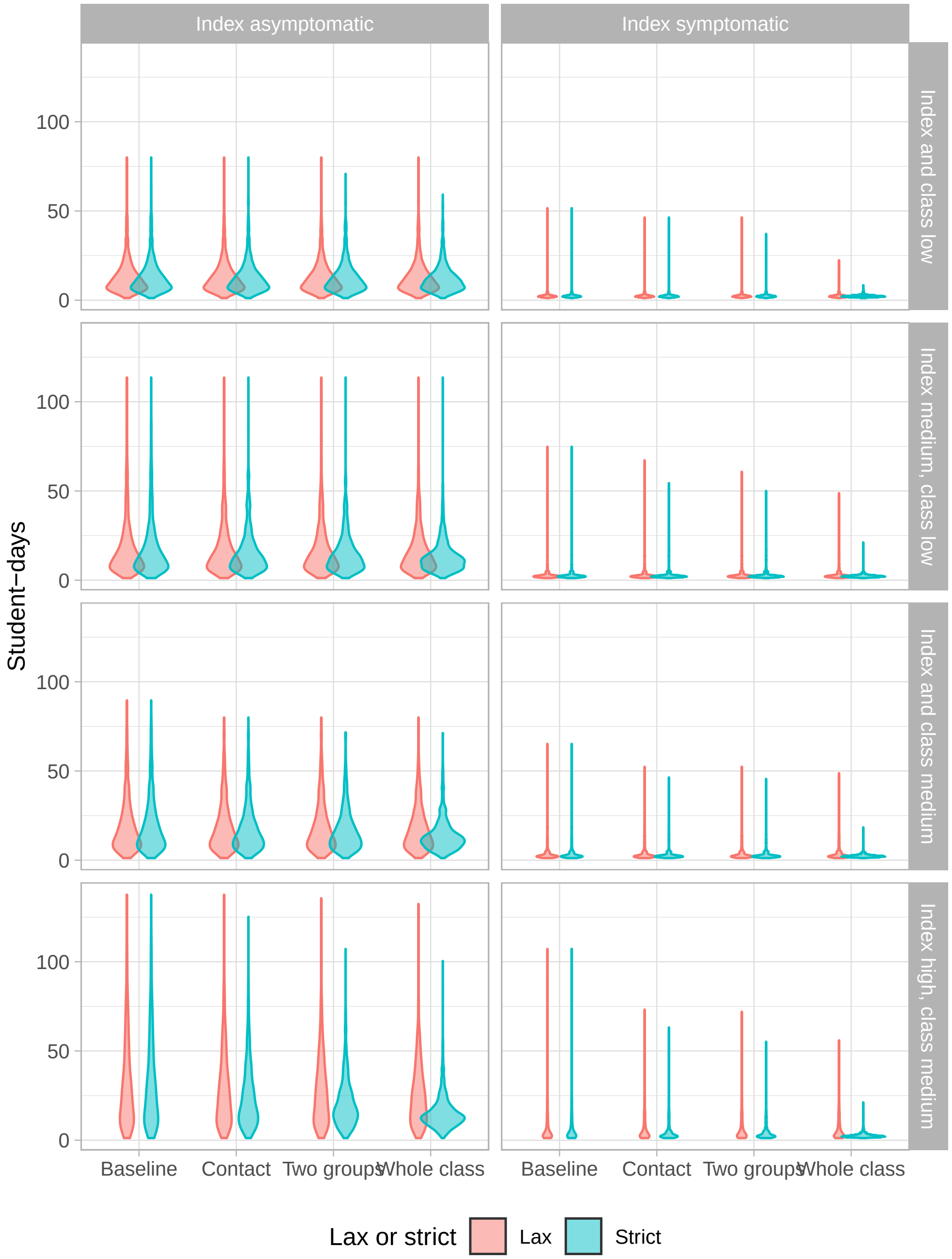

Fig E. Student-days of undetected (here, asymptomatic) infection in the high school protocols.

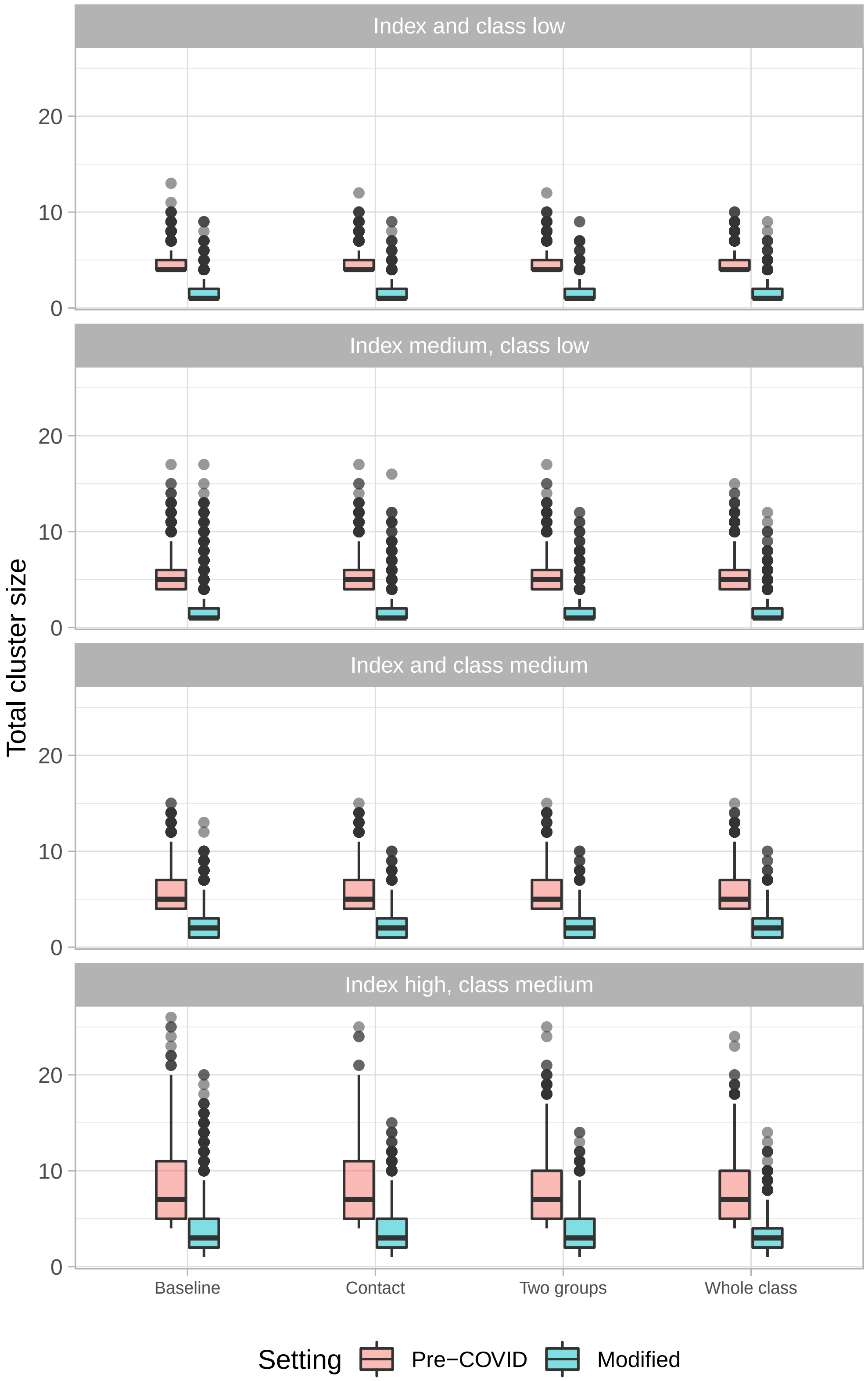

Fig F. Comparison of total number of infected students in a pre-COVID high school structure versus the modified plan we have studied here.

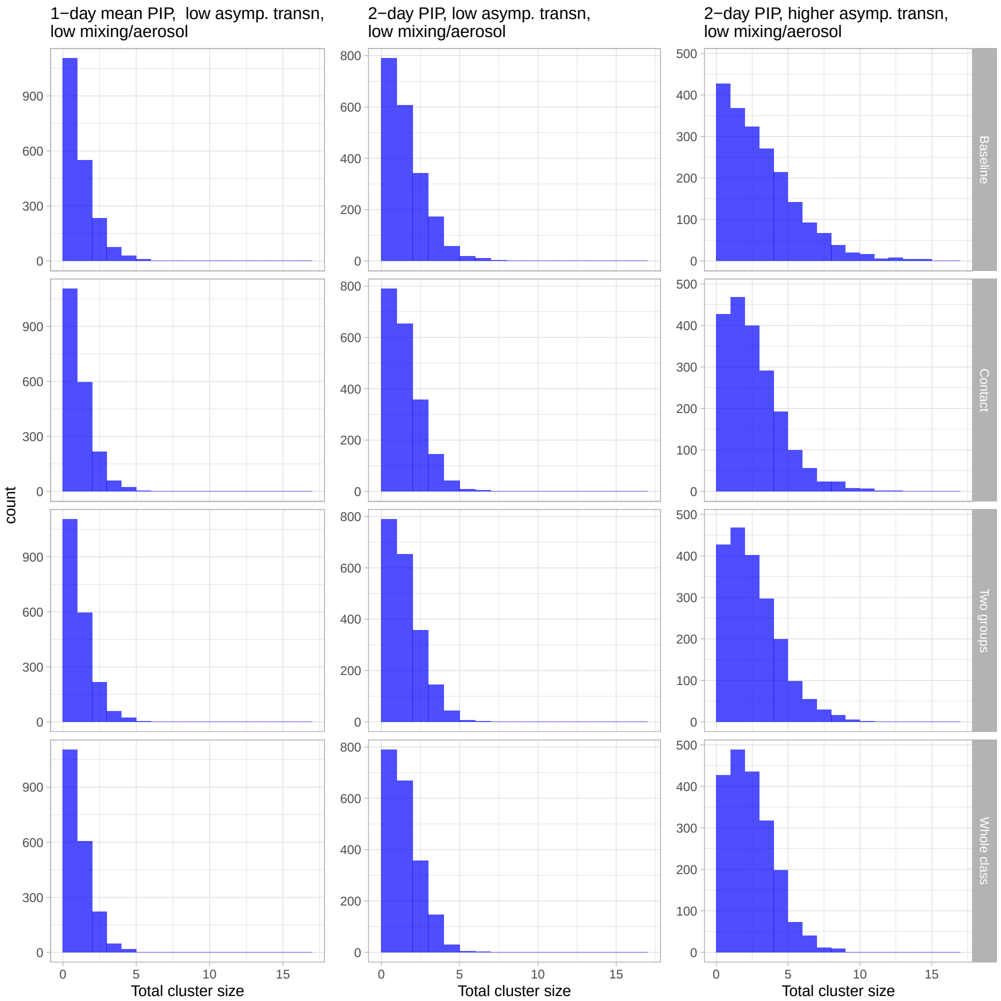

Fig G. Cluster sizes vary according to whether assumptions (1)-(3) hold. We show this progressively in this figure and the next, with the pre-symptomatic infectious period (PIP) (1 day in the left column, 2 days thereafter), the relative infectiousness of those who do not develop symptoms (0.2 times the baseline in the left two columns and 0.8 in the right column).

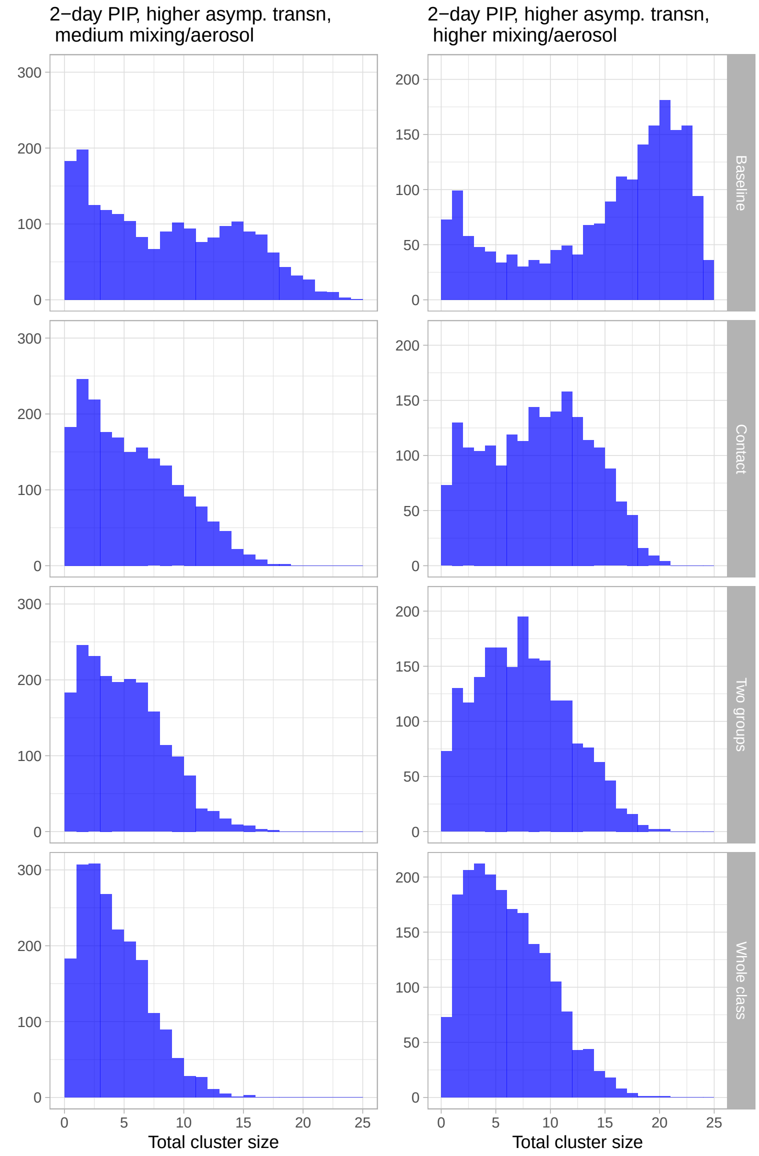

Fig H. Cluster sizes vary according to whether assumptions (1)-(3) hold. We show this progressively in this figure and the previous one, with the pre-symptomatic infectious period (PIP) (2 days here), the relative infectiousness of those who do not develop symptoms (0.8 here) and the relative transmission rate to individuals outside the close group of contacts compared to within - the mixing/aerosol parameter (0.25 in the left columns and 0.5 in the right column).

References

1. Gamma- RDocumentation;. https://www.rdocumentation.org/packages/Rlab/versions/2.15.1/topics/Gamma.

2. Gamma Distribution - MATLAB & Simulink;. https://www.mathworks.com/help/stats/gamma-distribution.html.

3. Zhao S. Estimating the time interval between transmission generations when negative values occur in the serial interval data: using COVID-19 as an example. Math Biosci Eng. 2020;17(4):3512–3519.

4. Tindale LC, Stockdale JE, Coombe M, Garlock ES, Lau WYV, Saraswat M, et al. Evidence for transmission of COVID-19 prior to symptom onset. eLife. 2020;9.

5. Danis K, Epaulard O, Bénet T, Gaymard A, Campoy S, Bothelo-Nevers E, et al. Cluster of coronavirus disease 2019 (Covid-19) in the French Alps, 2020. Clinical Infectious Diseases. 2020;81(1):179–182. doi:https://doi.org/10.1093/cid/ciaa424.
