# Supplementary material for: COVID-19 in schools: Mitigating classroom clusters in the context of variable transmission
Source: PLoS Comput Biol. 2021 Jul 8;17(7):e1009120. doi: 10.1371/journal.pcbi.1009120 (PMC8266060; doi:10.1371/journal.pcbi.1009120)
Supplement: Attachment — Submitted filename: response_to_PLOS_CompBio.pdf [file pcbi.1009120.s002.pdf]

Response to reviewer comments on PCOMPBIOL-D-20-01998

P. Tupper and C. Colijn

March 23, 2021

We thank the reviewers for their feedback on our work. Here we address each of their comments in turn.

Reviewer #1:

This paper looks at modelling COVID infections and possible interventions in the context of schools and the upset to education.

I have mixed feelings about this paper, but with a little work, I think I could be made publishable. Firstly, is this journal the right place for this paper? Being a computational journal I would have expected more discussion regarding the stochastic simulation. Further, it wouldn't be too difficult to wrap some theory around these simulations either from a stochastic point, using a chemical master equations approach and Stochastic Simulation Algorithm, or as a system of ODEs. I understand the authors want to consider the stochastic effects, but the mean-field averages over 1000 simulations should start to provide results that could be compared with an ODE approximation. Essentially, providing these comparisons would give me more confidence in knowing that a large stochastic matlab simulation is doing the right thing.

We have added a section in the Supplemental Material where we discuss more details of the simulation method.

In response to the reviewers query about why we didn't use an ODE model or the Master equation, there are a few reasons. As for ODEs, we consider classes that have a small number of students, so it doesn't make sense to consider number of infected and similar variables as continuous. Also, as noted by the reviewer, we are interested in stochastic effects, since it is the rarer events that are the most important for interventions, rather than the average ones. As for the Master equation, the version of it that we are familiar with is for Markov processes, and our model is not Markov. Since the latent period, the presymptomatic infectious

period, and the infectious period are all gamma distributed in our model, disease progression in an individual violates the Markov property. But perhaps the most important reason we used an individual-based stochastic model is that it allowed us to flexibly consider a wide variety of different interventions in the classroom. The plots that we generate from such simulations (such as what we show in figure 2) are directly interpretable by people without specialized training.

Definitely more analytical approaches could offer insights, but they were not what we pursued, given our desire to rapidly impact public health policy. However, to offer a little of what this reviewer is asking for, we compare some of our results with a simple analytically tractable model for the number of infected students in classroom clusters. This is now detailed in an additional section in the supplemental material.

But if the simulation is not the novelty, then perhaps the results are. However, we reach a sticking point again. Namely, the three results are: reduce contacts, provide better ventilation and, finally, testing is a great help. However, none of the insights are new, thus the authors should provide context for why their result is novel.

We don't agree that these are our main three results. Our major result as stated in the Abstract is "None of the mitigation protocols we modeled, initiated by a positive test in a symptomatic individual, are able to prevent large transmission clusters unless the transmission rate is low (in which case large clusters do not occur in any case). Among the measures we modeled, only rapid universal monitoring (for example by regular, onsite, pooled testing) accomplished this prevention." This was definitely not obvious. We were quite surprised that the protocols we considered based on testing symptomatic students were so ineffective, and we only turned to pooled testing when we learned this from our simulations. It was enough of a surprise that we got a lot of media exposure with interviews on TV and radio based on this work, since the prevailing opinion among public health officials in BC was that contact tracing of this sort would be effective, and pooled testing unnecessary.

Another product of our work is the code itself, which others can use. We were asked recently to model the effect of using rapid tests on staff at long term care homes for preventing COVID-19 outbreaks. We modified the code we developed for schools to suit the population structure and group sizes appropriate to long term care settings, and then used the results in this different setting to argue for changes in screening policy.

Overall, I hate to be negative as the paper is well written and I fully believe their results. Moreover, their introduction is an excellent review of current literature. However, at this time I feel that the article would be adding to the noise and rush of COVID publications, rather than boosting a signal that either provides a measurable impact, or a new idea that could tested. If the authors could

add further information regarding their mathematical rigour as to make the simulations feel less ad hoc and/or provide context for why their results are needed, then I would be happy to change my mind.

People are still using contact tracing as a cornerstone COVID-19 response. But the effects of contact tracing come too late. Our results illustrate the impact that mass testing (either pooled or otherwise) can have, and why. Mass testing can identify infectious individuals early enough to prevent clusters from occurring even in high-transmission circumstances. Furthermore, these circumstances can arise due to the index individual having a high transmission rate, and/or because of the environment in the setting. We're do not see that additional mathematical rigour could add more from a practical perspective.

Minor comments

Figure text is often too small to read. In many figures the "y" axis is not labelled and so it is hard to understand what we are reading and whether the distributions are comparable.

Good point. We've added a y-axis label to clarify what is shown (the count, as the relevant plots are all histograms). The ggplot2 stat binline plot that permits splitting the histograms according to the intervention does not also allow a numeric value to show the numbers, but y-axis values are comparable from panel to panel, and the overall scale is determined by the number of simulations making up the histogram.

The classroom parameters appear to be very specific to a particular school set up. How dependent are the results on this set up?

We actually consider an additional set-up: high schools, in the supplemental information. The results are qualitatively the same. We could of course consider many different possibilities, but already we feel that we present quite a lot of variations of the parameters as is; see the section Alternative Parameter Choices in the Supplemental Material. We have made the code for the simulations available so that researchers can try it with whatever settings they are interested in.

There are a few typographical errors throughout, for example lines 218 and 230.

Thanks, both are corrected. We've corrected several others we've spotted while making changes.

Reviewer #2:

This is largely subjective but I do not like the use of the word 'unfortunate', in the title and elsewhere, to describe large clusters. The assumption must be that these large clusters potentially lead to deaths after transmission from children to

older or more vulnerable people and I don't think the word unfortunate handles this in a sensitive way. Relatedly, the grammar in the title sounds very odd to me due to using the possesive "COVID-19's". Simply "Superspreading events of COVID-19 in schools: mitigating classroom clusters in the context of variable transmission" or "Large clusters of COVID-19 in schools: ..." avoids the word unfortunate and sounds grammatically less odd.

Yes, we take your point. We have changed the title to *COVID-19 in schools: mitigating classroom clusters in the context of variable transmission*.

A bit more detail on the Covid Écoles Québec data would be useful. Are the cluster sizes based only on testing symptomatic students? If so the cluster sizes are presumably underestimates. Given the qualitative nature of the comparisons being made, I don't think this is a big problem but a clear description would be useful.

We have added the following sentence to explain this:

> Cases were only detected through a PCR test after the appearance of symptoms, so the reported clusters are likely underestimated in size, and many exposures and smaller clusters will be missed altogether.

Some plots (histograms or density plots) or summary statistics (95% intervals), perhaps in the supplementary material, for the gamma distributions used for the latent period, PIP and infectious period would be useful. At first look, the Gamma(mu = 10, sigma = 5) for infectious period seemed very unlikely to me. It took me quite a long time to examine the distribution further because there is no further details in the paper and the default gamma distributions in R use scale and shape. My choice of software is my problem of course but as these assumptions largely drive the entire conclusion of the paper, making them easy to examine is a good thing.

In the main text we have added

> See the Supplemental Material for information about the parametrization and distribution of the latent period, the PIP, and the infectious period.

In the supplemental information we've now added a section explaining the parametrization of the gamma distribution, and histograms showing the distributions of the latent period, PIP, and infectious period.

In many of the figures the two columns of subplots are labelled no and yes. This would be much clearer if they were "Index symptomatic" and "Index asymptomatic" or just "symptomatic" and "asymptomatic".

Good suggestion. We have changed this throughout.

Line 218: "Symptomatic student are remain home and cannot" should be something like "Symptomatic students remain home and cannot"

Thanks, we fixed this.

I found the interplay between "baseline", "contact", "two groups is an outbreak", "whole class" and "lax", "strict" quite confusing. For example: in contact, "all the other students in their group are isolated". "Under a lax policy we assume that asymptomatic or presympomatic students are never told to isolate". I don't understand how these two things can occur at the same time or if they don't I don't understand the relationship between policies and protocols.

Sorry for the confusion. We've added more text explaining this, we hope more clearly. The paragraph describing the difference between lax and strict policies now reads:

> We did not explicitly simulate the number of new clusters that a cluster seeds through out-of-class social contacts (siblings, parents, teacher-teacher contact, after-school activities and so on). A measure of the risk of such "bridging" interactions is *asymptomatic student-days*, the total number of student-days when students are infectious, but are not isolating outside of the classroom. We assume that all symptomatic students are isolating outside the classroom, but whether asymptomatic students are depends on student behaviour, which is influenced by public health guidelines. We use the term *policy* to indicate the guidelines for student isolation outside the classroom, and its subsequent effect on student behaviour, (whereas we use *protocols* to refer to what happens in the classroom, as before.) Under a *lax* policy we assume that asymptomatic or presymptomatic students are not told to isolate (regardless of whether their group or class is shut down), and the number of asymptomatic student-days is just the total number of student-days of infectiousness without symptoms. Under a *strict* policy we assume that when a group or class is shut down all students in the group are told to isolate until they recover or receive a negative test result. So the asymptomatic student-days under this policy is the total number of days students are infectious but asymptomatic *before* their group or class is shut down.
