# Supplementary material for: COVID-19 in schools: Mitigating classroom clusters in the context of variable transmission
Source: PLoS Comput Biol. 2021 Jul 8;17(7):e1009120. doi: 10.1371/journal.pcbi.1009120 (PMC8266060; doi:10.1371/journal.pcbi.1009120)
Supplement: Attachment — Submitted filename: Second_response_to_PLOS_CompBio.pdf [file pcbi.1009120.s003.pdf]

Second response to reviewer comments on PCOMPBIOL-D-20-01998

P. Tupper and C. Colijn

May 9, 2021

We thank reviewer 1 for their additional comments. Here we address the comments in turn.

Reviewer #1:

My first comment was in regard to the amount of trust I can place in a computational simulation. I understand that they are focused on the stochastic features of the simulations. However, the ODE formulation was not intended to provide "accurate" results only provide confidence that the numerics are correctly implemented and doing what they are expected to be doing. I am happy for these results to be put in supplementary information, however, the results don't appear to compare well.

Specifically, the authors consider a simplified set up, which they say should match an analytical approach. Firstly, the text is not clear enough to reproduce how the results were produced, secondly, the left and right figures of (supplemental) Fig 2. don't appear to quantitatively correspond in the low transmission cases, where it is suggested that they should.

Since the authors cannot provide a simplified case that can be analytically checked, I have less confidence in the full simulation.

We think we understand better what the reviewer is asking for: a situation where the outputs of the model are known analytically, and so results from the simulation can be compared directly with an exact solution. We now provide a section in the Supplemental Material that explores such a situation: Analytical comparison in a simplified setting. We have omitted the section that existed previously.

The situation we consider is when only the index case can infect other students, and both the presymptomatic infectious period and the infectious period are deterministic. These changes were obtained by modifying three lines of code. Now the number of infected individuals in the class is 1 plus two independent binomial random variables, and so we can sample from this distribution using a short script independent of our model simulation code. We show the excellent agreement between samples from this distribution and results generated by our code. Furthermore, this serves to test the infection process generally, as the infections in the full simulations (i.e. the indirect infection simulation) use the same algorithm.

The second comment that has been ignored is that the y axes have still not been labelled with scales. The authors offer the response that "The ggplot2 stat binline plot that permits splitting the histograms according to the intervention does not also allow a numeric value to show the numbers, but y-axis values are comparable from panel to panel, and the overall scale is determined by the number of simulations making up the histogram." To which my response is simply don't use ggplot2 then. Without knowing the values of the data we cannot hope to be able to understand the authors results clearly. Moreover, the histogram can be normalised to produce a probability density, which is independent of the number of simulations.

The "values of the data" were, and remain, clearly indicated on the x axes in the relevant figures. The

notion that one "cannot hope" to compare distributions from stacked histograms – in the usual way that one interprets a histogram – without a numerical value showing the *counts* in the histograms is surprising to us. It is particularly surprising in light of the reviewer's appreciation of the fact that one can normalize distributions to produce a density (changing the y scale by a constant factor). Nonetheless, we have changed the visualizations, avoiding the style of 'stacking' histograms we had used (which is designed not to show the count numerically). The count scales on the y axes are now labelled numerically.

Equally one of my minor comments was ignored in that the text on many figures is still too small (supplemental figures too). As a rule of thumb the figure text should be no smaller than the caption.

We have resized the text and/or the figures at the reviewer's request, in the main text and the supplement. With reference to the main text, as the reviewer is surely aware, features such as font sizes relative to captions in the published version are determined by the journal's formats and by changes that may occur at the post-acceptance editorial stages. PLOS Computational Biology has a range of requirements for the figure format and dpi. If the reviewer finds that PLOS Computational Biology articles (main text) have figure fonts that are too small compared to their captions in the online format, this should be brought to the attention of the appropriate PLOS staff.

Finally, one of my comments asked for a comment of novelty and impact. The authors have supplied this within the reviewer rebuttal, but didn't add anything to the document? Specifically, I would like to see a section near the discussion which emphasises the impact that the work has had. This will encourage a reader to use the work and codes supplied by the authors.

Many journals explicitly prohibit claims of novelty: it is for the reader to decide what is novel or not. As for impact, one of the reasons for publishing in a journal is to reach the journal's audience, which may result in more impact. Does a paper have to have had its impact in large degree before it is published? These are surely editorial questions which the board of PLOS Computational Biology can discuss if they so choose. In our case, for the question of potential impact, if the reviewer doesn't think a paper on COVID-19 in classrooms that makes policy recommendations does not have potential impact, we are not sure what would make the reviewer happy.

As it happens, our work in preprint form has had impact. At the reviewer's request, we now cite evidence of this impact in a new final paragraph of the main text.
